# Delta Neutrophil Index as a New Early Mortality Predictor after Liver Transplantation

**DOI:** 10.3390/jcm12072501

**Published:** 2023-03-26

**Authors:** Jeongjun Lee, Sunyoung Son, Heeyoung Kim, Manki Ju

**Affiliations:** Department of Surgery, Gangnam Severance Hospital, Yonsei University College of Medicine, Seoul 06273, Republic of Korea

**Keywords:** DNI, survival predictor, liver transplantation

## Abstract

Background: Patients with liver disease display numerous defects of the immune system, so infection is a frequent complication of both acute and chronic liver disease. These infections are independently associated with poor outcomes after liver transplantation. Our objective was to evaluate the delta neutrophil index (DNI), a new inflammation marker, as a predictor of survival after liver transplantation (LT). Methods: This observational study retrospectively evaluated the records of 712 patients who underwent LT from January 2010 to February 2018. DNI was evaluated at pre-transplantation and 1, 7, 14, and 30 days after operation. Statistical analysis was performed using the T-test or chi-square test, and logistic regression analysis. Results: The mean MELD score was 16.7 ± 9.4 (0–48). There were 125 mortality cases (17.8%) after liver transplantation. Mean DNI was 1.61 at pre-transplantation, 3.94 one day after operation, 2.67 seven days after operation, 1.61 fourteen days after operation, and 1.64 thirty days after operation, respectively. In multivariate analysis, DNI seven and fourteen days after operation was revealed as an independent prognostic factor for mortality after liver transplantation (*p* = 0.040 and *p* < 0.0001). Conclusions: The DNI is a simple and reliable predictor of patient mortality after liver transplantation.

## 1. Introduction

Several factors influence graft outcomes after liver transplantation (LT) [1,2,3]. LT includes poor recipient condition, smaller graft volume (GV), and advanced donor age [4,5,6,7]. The Model for End-stage Liver Disease (MELD) score has been used to objectively quantify the severity of recipient disease and to prioritize organ allocation in patients awaiting deceased-donor LT (DDLT) [8].

Infection is a frequent complication of acute and chronic liver failure. Patients with liver failure have been shown to display numerous defects of the immune system, including impaired monocytes [9,10], neutrophil function [11,12,13], and complement deficiency [14]. A recent study on patients with cirrhosis suggested that pre-transplant culture-positive septic shock is associated with poorer outcomes in liver transplant patients with high acuity (MELD > 40) [15]. Although this study examined patients with chronic liver failure only, it demonstrated that severe pre-transplant infections may confer adverse effects, even after LT. However, among patients with acute liver failure (ALF), the impact of infection on survival remains unclear [16]. In addition, whether infections are independently associated with worse outcomes in patients with less severe acute liver dysfunction (e.g., acute liver injury without hepatic encephalopathy) remains unclear. Compared with earlier studies, substantial advances in critical care have occurred, including improvements in supportive care for patients with septic shock and acute respiratory distress syndrome [17,18]. Antibiotic prophylaxis is not universally adopted in critically ill patients admitted with acute liver dysfunction owing to the uncertainty of who might benefit from this practice and the potential increase in adverse effects and resistant organisms from routine antibiotic therapy. Therefore, it is of prognostic and potentially therapeutic importance to determine whether infections among critically ill patients with acute liver injury independently contribute to adverse outcomes.

Mortality rates, adjusted based on mortality predictions provided by prognostic score systems or independent variables, have been increasingly used to compare the quality of care provided by different intensive care units (ICUs) and hospitals. They are also used to evaluate the impact of new therapeutic options or organizational modifications as part of quality improvement initiatives [19].

The use of a specific automated blood cell analyzer, a recent technological advancement, allows rapid determination of the delta neutrophil index (DNI), which reflects the fraction of circulating immature granulocytes in the blood along with the complete blood count [20,21,22]. DNI has implications for the prevalence of overt DIC, bacterial isolation rate, and mortality in patients with suspected sepsis.

Herein, we evaluated the significance of the DNI as a prognostic marker of early mortality in patients with LT.

## 2. Materials and Methods

**Figure 1:**
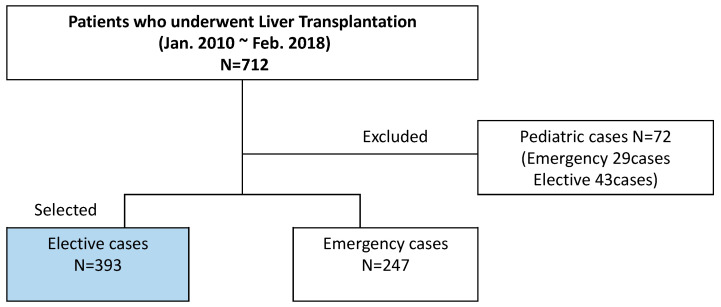
Schematic diagram of the patient selection process.

Clinical data of consecutive adult patients who underwent primary LT at our institution from January 2010 to February 2018 were extracted from our database and reviewed. Of the 712 patients, pediatric patients and patients undergoing emergency operation were excluded from the study group to control for confounding factors and to minimize the variations in patient characteristics. A total of 393 elective adult cases were selected (Figure 1). They were divided into two groups: those who experienced mortality within 30 days postoperatively [mortality (+)] and those who did not [mortality (−)].

The DNI was checked pre-transplantation and one, seven, fourteen, and thirty days after operation with other clinical variables.

The DNI was determined using an automatic cell analyzer (ADIVA 2120 Hematology system, Siemens Healthcare, Erlangen, Germany). The DNI was calculated using the following formula: DNI = [neutrophil and eosinophil subfraction measured in the myeloperoxidase (MPO) channel by cytochemical MPO reaction] − [polymorphonuclear subfraction measured in the nuclear lobularity channel by the reflected light beam].

IBM SPSS Statistics ver. 20.0 (IBM Co., Armonk, NY, USA) was used for all statistical analyses. Categorical variables were analyzed using chi-square tests or Fisher exact tests, and continuous variables were analyzed using Mann–Whitney U tests. Univariate and multivariate analyses were performed using logistic regression analysis. A value of *p* < 0.05 was considered statistically significant.

Overall survival was defined from the date of operation to the date of death or the last follow-up. Survival curves were plotted using the Kaplan–Meier method, and intergroup differences were assessed using the log-rank test.

Receiver operating characteristic (ROC) curves were generated to clarify the optimal DNI cutoff level for predicting mortality. The role of the DNI in predicting mortality was evaluated by the area under the ROC curve. The optimal cutoff level was determined by drawing a line connecting the points nearest to the left-upper corner. This method for determining the optimal cutoff level accounts for the trade-off between sensitivity and specificity over a continuous range.

## 3. Results

The characteristics of the study population are summarized in Table 1. There was a statistically significant difference in the transfusion amount, ICU duration, and application of continuous renal replacement therapy (CRRT) between the 30 days mortality (−) and (+) groups.

However, there was no statistically significant difference in the preoperative C-reactive protein (CRP) level and DNI between the two groups.

**Figure 2:**
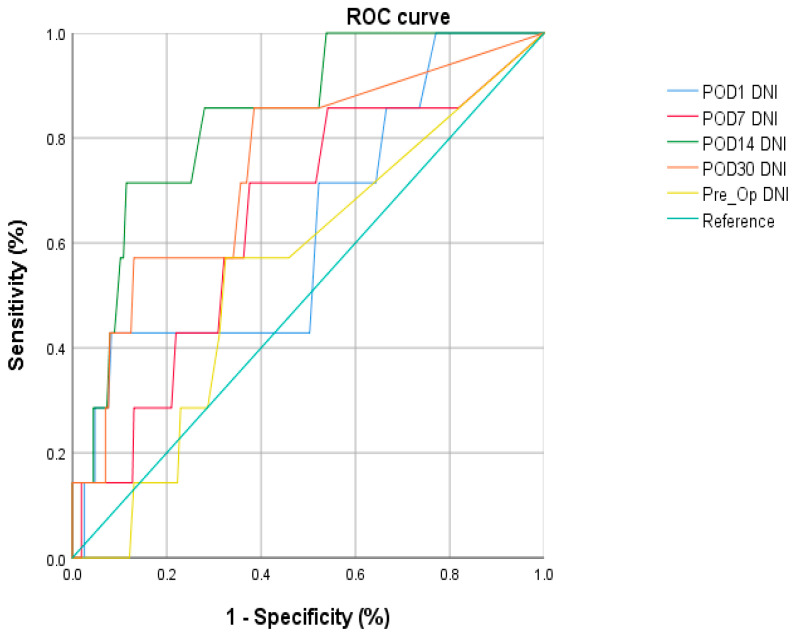
The optimal DNI cut-off level in POD #14 was 2.05 and this cut off level had a sensitivity of 81.8% and a specificity of 82.9%.

Guided by the receiver operating characteristic curve analysis, we chose the DNI at postoperative day (POD) 14 with a cut-off value of 2.05 to best differentiate survival. This cutoff level had a sensitivity of 81.8% and a specificity of 82.9% (Figure 2).

Table 2 shows the comparison of 30 days mortality according to the DNI level at POD 14 with a cut-off value of 2.05. Patients with POD 14 DNI ≥ 2.05 showed a statistically significant higher mortality rate within 30 days (DNI ≥ 2.05 = 12.2% vs. DNI < 2.05 = 0.6%; *p* = 0.008).

Patients with a DNI ≥ 2.05 on POD 14 had higher CRP levels in postoperative care. Operation time was longer in patients with a DNI ≥ 2.05 on POD 14 than in patients with a DNI < 2.05 on POD 14 (*p* = 0.017). Larger transfusion rates, longer ICU duration, and lower CRRT history were also found in the DNI ≥ 2.05 on POD 14 group.

Univariate and multivariate analyses including other factors showed that DNI ≥ 2.05 on POD 14 was the only mortality predictor (*p* = 0.001) (Table 3).

## 4. Discussion

DNI is a relatively new marker that is unfamiliar to many clinicians and has not been studied worldwide in contrast to other well-known markers such as procalcitonin [23], CRP [24], and ANC [25].

The use of a specific automated blood cell analyzer—a recent technological advancement—allows for the rapid determination of the DNI, which reflects the fraction of circulating immature granulocytes in the blood, along with the complete blood count (CBC). Though there have been numerous individual reports of DNI being far superior to the established inflammation markers for diagnosing infection and predicting mortality from infectious disease, whether this phenomenon holds true across all patients with different manifestations of infections (i.e., bacteremia, SIRS, sepsis, and septic shock), and whether DNI’s overall diagnostic accuracy is comparable to that of the other conventional markers, has not been thoroughly investigated. If the WBC count is within the normal range, it can be challenging to diagnose an infection [26].

A previous study by Park et al. revealed that a DNI > 6.5% was a good diagnostic marker of severe sepsis and septic shock within the first 24 h after intensive care unit admission [27]. Previous studies have proposed potential mechanisms to explain this rapid and early release of immature granulocytes in sepsis.

One such study describes the rapid expansion of circulating neutrophils to compensate for the loss of active neutrophils due to the massive consumption and destruction of mature cells in severe inflammation [28].

Immature granulocytes are one of the biomarkers that predict post-transplant prognosis. As most immature granulocytes are increased post-transplantation, early increases could be regarded as a result of inflammation, such as acute graft rejection or surgical trauma [29,30]. Although there are not many studies on DNI in transplant patients, the prognosis of transplantation recipients with increased DNI is poor [30,31]. In addition, the DNI level is not only increased in infectious condition but also increased in inflammatory conditions, such as pre-eclampsia [32].

Hence, we propose the use of the DNI, which can be determined rapidly, easily, and inexpensively, to assess severity in such patients. The purpose of the study was to evaluate the significance of the DNI as a prognostic marker of early mortality in patients who underwent LT. The study population consisted of 393 adult patients who underwent primary LT and were divided into two groups: 30 days mortality (−) and (+) groups. The DNI was checked pre-transplantation and one, seven, fourteen, and thirty days after operation, along with other clinical variables. The results showed that there was a statistically significant difference in the transfusion amount, ICU duration, and application of continuous renal replacement therapy (CRRT) between the two groups. However, there was no statistically significant difference in the preoperative CRP level and DNI between the two groups. The receiver operating characteristic curve analysis found that DNI at POD 14 with a cut-off value of 2.05 was the best differentiation of survival. The results showed that patients with POD 14 DNI ≥ 2.05 had a statistically significant higher mortality rate within 30 days compared to those with DNI < 2.05. The study concludes that DNI at POD 14 with a cut-off value of 2.05 is a significant predictor of early mortality in patients who have undergone LT.

Most patients with high MELD scores have an inflamed status. Therefore, these patients show high DNI, CRP, and other inflammatory markers [33,34,35]. The outcomes were also unfavorable and the 3-month and 1-year overall survival rates of recipients with MELD scores ≥ 38 were 75.5% and 68.2%, respectively [36]. Thus, we attempted to increase the homogeneity of the study population by excluding high-MELD (>35) emergency deceased donor transplantations, as these patients were generally worse before transplantation.

Opportunistic infections after transplantation are generally absent during the first month after transplantation since the full effect of immunosuppression is not yet present. Infections such as viremia and candidemia in this period are generally donor-derived or recipient-derived, or they are associated with technical complications of surgery [37]. Therefore, there is little effect on DNI during the early period after transplantation.

This study has several limitations. First, due to its retrospective design and the inclusion of a patient cohort derived from a single, tertiary, academic hospital, it was difficult to control for confounding factors, thus increasing the risk of selection bias. However, we used a critical pathway that was prospectively performed with a standardized and predetermined protocol.

Second, we could not accurately assess the long-term clinical outcomes. Third, heparin has been shown to inhibit neutrophil activation and induce the aggregation and apoptosis of neutrophils [23].

Unfortunately, our institution no longer uses a cell analyzer that automatically calculates the DNI. Therefore, further follow-up or new prospective studies are impossible.

## 5. Conclusions

The results showed that the DNI at postoperative day 14 with a cut-off value of 2.05 was a significant prognostic marker of early mortality, with higher mortality rates observed in patients with a DNI ≥ 2.05. This finding highlights the potential utility of the DNI as a new early mortality predictor after liver transplantation, which could provide valuable information for clinical decision-making and improve the management of patients with liver transplantation. Further studies with larger sample sizes and long-term follow-up are needed to validate these findings and establish the DNI as a routinely used tool for prognostication after liver transplantation.

## Figures and Tables

**Table 1:** The characteristics of the study population.

		30 Days Mortality (−)		30 Days Mortality (+)		*p*-Value
Age		54.1 ± 8.4	17~72	51.0 ± 9.6	33~64	0.239
Sex	Male	289	76.05%	8	61.5%	0.8
	Female	91	23.95%	5	38.5%	
MELD		13.5 ± 7.5	5~42	20.7 ± 11.1	9~43	0.002
OP time		11.8 ± 2.1	7.2~23	13.1 ± 3.0	8~19.3	0.05
Transfusion		5.83 ± 9.16	0~97	29.4 ± 25.3	2~72	<0.0001
ICU duration		4.58 ± 2.73	0~24	8.33 ± 4.5	1~16	<0.0001
Renal replacement	Yes	2	0.6%	1	8.3%	0.001
	No	378	99.4%	12	91.8%	
Pre OP CRP		11.1 ± 22.7	0~216	21.14 ± 21.6	0.8~55.4	0.19
Pre OP DNI		1.0 ± 2.51	0~36.9	2.1 ± 2.3	0~7.4	0.18
POD 7 CRP		18.3 ± 28.3	0.95~168.8	41.6 ± 57.2	1.6~181.7	0.024
POD 7 DNI		2.1 ± 2.6	0~32.4	3.9 ± 2.9	0~8.2	0.021
POD 14 CRP		15.5 ± 22.7	0~130.5	88.9 ± 80.2	7.5~212	<0.0001
POD 14 DNI		1.1 ± 1.5	0~12.5	8.2 ± 13.7	0.5~47.3	<0.0001

MELD, Model for End-stage Liver Disease; OP, operation; ICU, intensive care unit; CRP, C-reactive protein; DNI, delta neutrophil index; POD, postoperative day.

**Table 2:** The comparison of 30 days mortality according to the DNI level at POD 14 with a cut-off value of 2.05.

		DNI ≥ 2.05 on POD 14		DNI < 2.05 on POD 14		*p*-Value
Age		53.3 ± 9.5	17~71	54.2 ± 8.1	17~72	0.42
Sex	Male	44	60.2%	253	79.5%	<0.0001
	Female	29	39.8%	65	20.5%	
MELD		16.4 ± 9.1	6~43	13.1 ± 7.2	5~42	0.001
OP time		12.4 ± 2.0	9~19.3	11.7 ± 2.2	7.2~23	0.017
Transfusion		10.7 ± 14.4	0~72	5.5 ± 9.2	0~97	<0.0001
ICU duration		5.9 ± 3.4	2~17	4.4 ± 2.6	0~24	<0.0001
Renal replacement	Yes	2	2.7%	1	0.3%	0.033
	No	71	37.3%	317	99.7%	
Pre OP CRP		12.3 ± 20.9	0.2~142.6	11.2 ± 23.2	0.2~216.2	0.74
POD 7 CRP		30.7 ± 43.2	2.4~181.7	16.3 ± 25.2	0.95~168.8	0.006
POD 14 CRP		34.6 ± 45.7	0.7~212.03	13.3 ± 21.3	0.4~130.45	<0.0001
30 days mortality		9/73	12.3%	2/318	0.6%	0.008

DNI, delta neutrophil index; POD, postoperative day; MELD, Model for End-stage Liver Disease; OP, operation; ICU, intensive care unit; CRP, C-reactive protein.

**Table 3:** Univariate and multivariate analyses including other factors showed that DNI ≥ 2.05 on POD 14 was the only mortality predictor (*p* = 0.001).

Variable	Univariate	Multivariate	
	*p*-Value	*p*-Value	OR (95% CI)
Age	0.83		
Sex	0.95		
Transfusion	0.99		
CRRT history	0.48		
MELD	0.26	0.064	1.06 (0.99~1.15)
OP time	0.36		
DNI ≥ 2.05 on POD 14	0.99	0.001	31.55 (3.83~260.3)

OR, odds ratio; CI, confidence interval; CRRT, continuous renal replacement therapy; MELD, Model for End-stage Liver Disease; OP, operation; DNI, delta neutrophil index; POD, postoperative day.

## Data Availability

Not applicable.

## Abbreviations

ALF—acute liver failure; CRP—C-reactive protein; CRRT—continuous renal replacement therapy; DDLT—deceased-donor liver transplantation; DNI—delta neutrophil index; GV—graft volume; ICU—intensive care unit; LT—liver transplantation; MELD—Model for End-stage Liver Disease; POD—postoperative day.
